# Loss of Secreted Frizzled-Related Protein 4 Correlates with an Aggressive Phenotype and Predicts Poor Outcome in Ovarian Cancer Patients

**DOI:** 10.1371/journal.pone.0031885

**Published:** 2012-02-21

**Authors:** Francis Jacob, Kristjan Ukegjini, Sheri Nixdorf, Caroline E. Ford, Jake Olivier, Rosmarie Caduff, James P. Scurry, Rea Guertler, Daniela Hornung, Renato Mueller, Daniel A. Fink, Neville F. Hacker, Viola A. Heinzelmann-Schwarz

**Affiliations:** 1 Translational Research Group, University Hospital Zurich, Zurich, Switzerland; 2 Gynaecological Cancer Group, Lowy Cancer Research Centre, Prince of Wales Clinical School, Faculty of Medicine, University of New South Wales, Sydney, Australia; 3 Wnt signaling and Metastasis Group, Lowy Cancer Research Centre, Prince of Wales Clinical School, Faculty of Medicine, University of New South Wales, Sydney, Australia; 4 Biostatistics Group, Lowy Cancer Research Centre, Prince of Wales Clinical School, Faculty of Medicine, University of New South Wales, Sydney, Australia; 5 Institute of Clinical Pathology, University Hospital Zurich, Zurich, Switzerland; 6 Hunter Area Pathology Services, John Hunter Hospital, University of Newcastle, Callaghan, Australia; 7 Department of Obstetrics and Gynecology, University of Schleswig-Holstein, Lubeck, Germany; 8 Department of Gynecology and Obstetrics, Spital Limmattal, Zurich, Switzerland; 9 Department of Gynecology, University Hospital Zurich, Zurich, Switzerland; 10 Gynaecological Cancer Centre, Royal Hospital for Women, Sydney, Australia; King Faisal Specialist Hospital & Research Centre, Saudi Arabia

## Abstract

**Background:**

Activation of the Wnt signaling pathway is implicated in aberrant cellular proliferation in various cancers. In 40% of endometrioid ovarian cancers, constitutive activation of the pathway is due to oncogenic mutations in β-catenin or other inactivating mutations in key negative regulators. Secreted frizzled-related protein 4 (SFRP4) has been proposed to have inhibitory activity through binding and sequestering Wnt ligands.

**Methodology/Principal Findings:**

We performed RT-qPCR and Western-blotting in primary cultures and ovarian cell lines for SFRP4 and its key downstream regulators activated β-catenin, β-catenin and GSK3β. SFRP4 was then examined by immunohistochemistry in a cohort of 721 patients and due to its proposed secretory function, in plasma, presenting the first ELISA for SFRP4. SFRP4 was most highly expressed in tubal epithelium and decreased with malignant transformation, both on RNA and on protein level, where it was even more profound in the membrane fraction (p<0.0001). SFRP4 was expressed on the protein level in all histotypes of ovarian cancer but was decreased from borderline tumors to cancers and with loss of cellular differentiation. Loss of membrane expression was an independent predictor of poor survival in ovarian cancer patients (p = 0.02 unadjusted; p = 0.089 adjusted), which increased the risk of a patient to die from this disease by the factor 1.8.

**Conclusions/Significance:**

Our results support a role for *SFRP4* as a tumor suppressor gene in ovarian cancers *via* inhibition of the Wnt signaling pathway. This has not only predictive implications but could also facilitate a therapeutic role using epigenetic targets.

## Introduction

Epithelial ovarian cancer (EOC) is the fifth most common cause of death from all cancers occurring in women and the leading cause of death from gynaecological malignancies. Over 75% of women present with locally advanced or disseminated disease, typically characterized by a gradual invasion of the surrounding organs and, in high stage cases, of the peritoneal cavity. Survival has changed little since the early 1980s despite new chemotherapeutical drugs. The survival rate of three-quarters of patients presenting with widespread metastatic disease is only around 20% [1].

This poor overall prognosis results from a lack of early symptoms and early diagnosis, ineffective therapy for advanced disease, resistance to platinum-based chemotherapies and from limited understanding of the early-initiating events and early stages of ovarian cancer development. A major challenge remains the identification of oncogenic ovarian cancer pathways to aid in diagnosis, as prognostic indicators and as targets for new therapeutic strategies [2]. Many groups, including our own, have utilized array-based genome-wide discovery platforms to identify aberrant mRNA expression and somatically acquired DNA sequence variants or mutations to determine the molecular changes underlying the development of ovarian cancer, as a first step to identify molecular markers with potential clinical utility [3], [4].

Using this technology, members of the Wnt signaling pathway have been implicated in ovarian carcinogenesis, as having the potential for diagnostic, prognostic and therapeutic targets [5], [6]. The Wnt signaling pathway is highly conserved throughout animals and mediates a variety of cellular functions including cell polarity, tissue patterning, control of cellular proliferation and development of neoplasia [7], [8]. Wnt proteins are secreted, cysteine rich signaling molecules with conserved structures. Nineteen Wnt proteins have been identified and linked to various stages of human development and carcinogenesis, including cancers of the breast, lung, colon, ovaries and skin [9], [10], [11], [12], [13], [14]. The Wnt proteins signal *via* Frizzled receptors through a number of different but interconnected signaling pathways, including the Wnt/Ca^2+^, β-catenin and planar-cell polarity pathways [15], [16], [17]. In general, the Wnt family is classified based on ligand and receptor involvement into the canonical/β-catenin pathway and the β-catenin independent/non-canonical pathway. Interestingly, non-canonical Wnt signaling can antagonize canonical Wnt signaling, and may represent a novel pathway to target cancers driven by canonical Wnt signaling [18]. Downstream target genes of the Wnt/β-catenin/TCF signaling pathway have been identified as being crucial for ovarian epithelial cell transformation, and were upregulated in all endometrioid ovarian cancers with Wnt pathway defects [19], [20]. Several other studies supported this observation, reporting overexpression of cyclin D1 in ovarian cancers carrying β-catenin mutations [21], [22], [23], [24].

Secreted frizzled-related proteins (SFRPs) are extracellular inhibitors of Wnt signaling that act by binding directly to Wnt ligands [25] or to Frizzled receptors [26]. Frizzled receptors are found exclusively at the plasma membrane, located at the surface of Wnt-responsive cells. In recent years, numerous reports have described epigenetic silencing of these canonical Wnt signaling antagonists in various human cancers, suggesting they may function as tumor suppressors [27]. In ovarian cancer, *SFRP1* was the first family member reported to be hypermethylated and silenced in ovarian cancer cell lines and patient specimens but not in normal controls, suggesting a potential role as a tumor suppressor [28]. Promoter hypermethylation of *SFRP2* and *SFRP5* was subsequently also found in ovarian cancer [29]. A recent study reported loss of *SFRP5* expression to be associated with both progression of ovarian carcinogenesis and chemotherapy resistance [29].

As we had previously identified *SFRP4* to be aberrantly expressed at the RNA level in a large transcriptional profiling experiment of ovarian cancer patients (unpublished data), here we investigate for the first time SFRP4 RNA and protein expression in 725 patients using reverse transcription quantitative polymerase chain reaction (RT-qPCR), Western-blot, immunohistochemistry (IHC) and capture enzyme-linked immunosorbent assay (ELISA) in primary cultures, ovarian cell lines, ascites, tissue and plasma.

## Methods

### Clinicopathological patient cohort

Ethical approval and written informed consent was granted at three different sites in Switzerland, Germany and Australia: 1. Department of Gynaecology, University Hospital Zurich and Department of Gynaecology and Obstetrics, Spital Limmattal, Zurich (SPUK, StV06/2006, to V.H.S.); 2. Department of Gynaecology and Obstetrics, University of Schleswig-Holstein (Ethics Committee of the University of Schleswig-Holstein, Campus Lubeck; to D.H.); and 3. Gynaecological Cancer Centre, Royal Hospital for Women, Sydney (HREC 08/09/17/3.02, to V.H.S.). Archival tissue from 721 patients within the Swiss Cohort with normal, benign and ovarian/tubal/peritoneal or endometrial cancers were included in tissue microarrays (281 benign diagnoses, 440 cancers), with the majority of cancers being of ovarian origin (69.8%; Table 1). Haematoxylin & Eosin (H&E) stained sections of each sample from both the Swiss and Australian Cohorts were reviewed by a pathologist specialized in gynaecological pathology (R.C. for Swiss Cohort; J.S. for Australian Cohort) and areas corresponding to tumor/benign tissue marked. Tissue core biopsies of 1.0 or 2.0 mm were incorporated into medium-density tissue microarrays (Beecher Instruments, Silver Spring, MD, USA). Each patient was represented by two cores sampled from different areas of the tumor. Sections from each array were H&E stained to confirm the inclusion of the selected tissue in each core, and patients with unclear or mixed histologies excluded. All clinicopathological patient data such as FIGO stage, grade, residual disease, presence of ascites, past and present medical illness, ultrasonographic findings and outcome data were stored in a specially designed in-house database (PEROV) based on Microsoft Access (unpublished; Microsoft, Seattle, USA). Patients with a past history of cancer or inflammatory/autoimmune diseases were excluded from this study.

**Table 1:** Clinicopathological characteristics of the patient cohort.

		Patient cohort (n = 721)
**BENIGN**		**281 (39%)**
**Healthy controls**		54 (19.2%)
	Tube	12
	OSE	18
**Endometriosis**		69 (9.6%)
**Benign tumors**		158 (56.2%)
**INVASIVE/NONINVASIVE TUMORS**		**440 (61%)**
**Borderline Tumors**		86 (19.5%)
**Cancers**		354 (80.5%)
**Ovarian cancers**		247 (69.8%)
	Serous	117 (47.4%)
	Endometrioid	49 (19.8%)
	Clear Cell	49 (19.8%)
	Transitional Cell	7 (2.8%)
	Mucinous	25 (10.1%)
**Type I cancers**		223 (50.7%)
**Type II cancers**		167 (38.0%)
**Endometrial cancers**		40 (11.3%)
**Others**		67 (18.9%)

Our plasma cohort was extended by 52 patients (German Cohort) for the purpose of facilitating a larger endometriosis patient group. Blood samples were collected in EDTA blood tubes (BD Vacutainer®, BD Diagnostics, Franklin Lakes, NJ, USA) prior to surgery and stored on ice until further processing. Samples were processed within 3 h by centrifugation of 3’000 g for 10 min at 4°C and plasma stored at −80°C.

### Cell culture

Cell line SKOV3 (serous ovarian cancer) was cultured in RPMI 1640 medium containing Penicillin/Streptomycin (Pen/Strep), 10% fetal calf serum (FCS) and L-glutamine (L-Glut). TOV112D (endometrioid ovarian cancer) and TOV21G (clear cell ovarian cancer) were cultivated in DMEM+Pen/Strep+10% FCS+L-Glut. Normal human ovarian surface epithelial cells (HOSE6-3) were cultured in Medium 199/MCDB 105 (1∶2) containing Pen/Strep+10% FCS+L-Glut. All cancer cell lines were derived from ATCC (www.atcc.org), HOSE6-3 was a gift from the Garvan Institute of Medical Research, Sydney, Australia.

### Primary cultures

Primary cultures were collected during surgery or repeatedly during paracentesis required for chemoresistant progressive disease (Australian Cohort). Ovarian cancer cultures derived during paracentesis were taken from the cell pellet generated after centrifugation of ascites at 4°C with 3’000 g. Tubal cells for culture were collected using a cytobrush at the fimbrial end of the tube immediately after prophylactic bilateral salpingo-oophorectomy. The two tubal cell lines used in this publication were derived from two patients undergoing risk-reducing surgery for *BRCA1* mutation status (Tube 1) and strong family history of ovarian/breast cancer (Tube 2), where written informed ethical consent was granted (HREC 08/09/17/3.02, to V.H.S.). After collection, cultures were instantly stored in DMEM and transferred to the laboratory for cultivation. Primary cultures were either grown in DMEM+Pen/Strep+10% FCS+L-Glut (ovarian cancer cell lines) or Medium 199/MCDB 105 (1∶2) containing Pen/Strep+10% FCS+L-Glut (normal tubal cell lines) until confluent. The second passage of each culture was used for the experiments.

### RT-qPCR

RT-qPCR was performed according to MIQE guidelines Cells were grown in 6-well plates (NUNC, Thermo Fisher Scientific, Roskilde, Denmark) to a confluency of 60%, washed with 1× DPBS (Gibco, Invitrogen Australia Pty Ltd, Mulgrave) and total RNA extracted (NucleoSpin RNAII kit, Macherey&Nagel, Duren, Germany). RNA concentration was measured using the NanoDrop ND-1000 spectrophotometer (Thermo Fisher Scientific, Roskilde, Denmark), the integrity confirmed by agarose gel electrophoresis (1.7% agarose gel) and a ratio of optical density of 260/230 nm≈2.1 (2.0 to 2.2) and 260/280≈2.0 (1.8 to 2.2) selected as inclusion criteria. QPCR was performed on three reference genes as well as the target gene, *SFRP4* using 500 ng reverse transcribed RNA in a total volume of 20 µl (iScript Reverse Transcription Supermix, Bio-Rad Laboratories Pty Ltd, Gladesville, Australia). Primers for reference genes were selected due to their stable expression: TATA box binding protein [30] forward 5′-TGCACAGGAGCCAAGAGTGAA-3′ and reverse 5′-CACATCACAGCTCCCCACCA-3′; beta-glucuronidase [31] forward 5′-AGCCAGTTCCTCATCAATGG-3′ and reverse 5′-GGTAGTGGCTGGTACGGAAA-3′; succinate dehydrogenase complex, subunit A [32] forward 5′-TGGGAACAAGAGGGCATCTG-3′ and reverse 5′-CCACCACTGCATCAAATTCATG-3′; and *SFRP4* forward 5′-ACACCCTCTTAAGCAGCACCAG-3′ and reverse 5′-AGGGTGGATGTCCTGGGAAGTAAG-3′ [33] (Sigma-Aldrich Pty Ltd, Castle Hill, Australia). QPCR performed on the Stratagene Mx3005® (Integrated Sciences Pty Ltd, Chatswood, Australia) using 96-well microtitre plates (Bio-Rad Laboratories (Pacific) Pty Ltd, Gladesville, Australia) and the SensiFAST™ SYBR lo-ROX Kit (Bioline (Aust) Pty Ltd, Alexandria, Australia) with low ROX as the fluorescence reference dye. Optimal reaction conditions were obtained by 2× SensiFAST™ SYBR mix, 400 nM specific sense primer, 400 nM specific antisense primer, RNase/DNase-free water and 25 ng cDNA template up to a final volume of 20 µl. Amplifications were performed starting with 30 sec enzyme activation at 95°C followed by 40 cycles of denaturation at 95°C for 5 sec and annealing/extension at 60°C for 30 sec. A melting curve was subsequently produced at 65–95°C. All samples and negative controls were amplified in triplicate and the mean of baseline-corrected normalized fluorescence signals (dRn) obtained for further calculations. Quantification cycle (Cq) values of our reference genes were combined in a geometric mean for each sample [32] and subtracted from *SFRP4* expression: ΔCq = Cq*_SFRP4_*-Cq_Ref_. To relatively quantify *SFRP4* expression (R) in all studied cell lines, HOSE6-3 was selected as our control and expressions of other lines were calculated as a ratio compared to HOSE6-3 as follows: R = 2^−[ΔCqSFRP4- ΔCqHOSE6-3]^.

### Western-blot analysis

Ascites was collected during paracentesis from two ovarian cancer patients at two consecutive time points, three months apart. Tubulin was used as a loading control for cell lines and anti-beta-2-microglobulin for the patient ascites protein extracts. Aliquots of 30 µg were combined with NuPAGE® LDS sample and reducing buffers (Invitrogen Australia Pty Ltd, Mulgrave) and boiled before loading onto sodium dodecyl sulfate (SDS)-polyacrylamide gels. All gels were electrically transferred to PVDF membranes before being blocked for 1 h at RT in 0.01% TBS/Tween containing 3% non-fat milk powder (TBS/Tween with non-fat milk). Membranes were incubated with primary antibodies overnight at 4°C in TBS/Tween with non-fat milk and then washed three times for 5 min in TBS/Tween. Visualization of proteins was performed *via* the addition of a secondary antibody conjugated to horseradish peroxidase (HRP), which was incubated for 1 h at RT in TBS/Tween with non-fat milk. Membranes were washed three times for 10 min in TBS-Tween, incubated in ECL and developed with hyperfilm. Scanning and quantification of signal intensities was performed using a Bio-Rad GS-800 densitometer with Quantity One software (Hercules, CA, USA). Antibodies were used at the following dilutions: SFRP4 (Abnova, #6424-A01): 1∶1’000, activated β-catenin (Millipore, #05-665) 1∶1’000, β-catenin (Santa Cruz Biotechnology, #SC-7963), GSK3β (Sigma, #G7914) 1∶1’000, anti-beta-2-microglobulin (Sigma, #WH0000567M1, 1∶1’000). All secondary antibodies were from DAKO (Dako Australia Pty Ltd, Botany, Australia) and were used at the following dilutions: goat anti-rabbit, 1∶5’000 and goat anti-mouse, 1∶5’000.

### Immunohistochemistry

For the detection of SFRP4 expression in various tissues, tissue microarray slides were analyzed using the Ventana Benchmark automated staining system (Ventana Medical Systems, Tucson, Arizona, USA). For antigen retrieval, slides were heated with cell conditioning solution for 1 h (CC1; Tris-based buffer with slightly alkaline pH) using a standard protocol. Slides were incubated for 1 h with primary mouse polyclonal anti-human SFRP4 antibody (1∶30; Abnova, Taipeh, Taiwan). Detection was carried out using the UView HRP system (Ventana Medical Systems, Tucson, Arizona, USA). Negative controls omitted the primary antibody, and a positive and negative control tissue for each antibody was identified from electronic Northern blot data or the published literature. Counterstaining was performed with hematoxylin and 1% acid alcohol. Immunostaining was scored as percentage and intensity of SFRP4 expression in various cellular compartments (membrane, cytoplasm, nucleus). Scoring was independently assessed by two researchers and discrepancies resolved by consensus. SFRP4 expression from all cores from one patient was averaged.

### ELISA

Serum SFRP4 concentrations were determined by an in–house developed Sandwich ELISA. Immunoplates (96-well NUNC MaxiSorp; Thermo Fisher Scientific, Roskilde, Denmark) were coated with a capturing mouse polyclonal antibody raised against a partial recombinant human SFRP4 protein (#H00006424-A01, Abnova, Taipei City, Taiwan) and diluted 1∶250 in 0.1 M phosphate buffer pH 7.2. Coating and incubation were performed overnight at 4°C. Recombinant protein, primary and secondary antibodies were diluted in PBS and plates washed three times with 0.05% (v/v) Tween 20 (Sigma-Aldrich Chemie GmbH, Buchs, Switzerland). Plates were blocked with PBS containing 5% (w/v) standard commercial available milk powder for 40 min at 37°C and washed three times. After blocking of unspecific binding sites a standard curve was developed by using recombinant SFRP4 protein (#H00006424-Q01, Abnova, Taipei City, Taiwan) ranging from 3’200 ng/ml to 12.5 ng/ml. Undiluted plasma samples were incubated in duplicate for 1 h at RT. Bound SFRP4 was detected using rabbit polyclonal primary antibody to human SFRP4 at RT for 60 min (Dr. R. Friis, University of Berne, Switzerland). Plates were washed three times followed by incubation with secondary polyclonal anti-mouse antibody conjugated to rabbit horseradish peroxidase (1∶1’000, Abcam, Cambridge, UK) for 60 min at RT. After five washing steps, chromogen TMB (Sigma-Aldrich Chemie GmbH, Buchs, Switzerland) was applied as a substrate and the peroxidase reaction stopped at 30 min by an equal volume of 2 M sulfuric acid. Optical density was measured using a 450 nm ELISA reader (Tecan Spectrafluor Plus, Tecan, Mannedorf, Switzerland).

### Statistical analysis

Whilst SFRP4 protein expression in tissues was initially quantified as cytoplasmic, membrane and nuclear staining, numbers were often too low for further statistical analyses of individual histological subgroups. Therefore, SFRP4 expression was combined, independent of the location of staining, and was called overall “SFRP4 expression” (arbitary units). SFRP4 expression in both tissue and plasma was initially described using boxplots for various diagnosis groupings. A normal quantile plot for SFRP4 expression in plasma was indicative of positive skewness making any subsequent analyses requiring a normal distribution assumption questionable. However, the variance stabilizing logarithmic transformation ameliorated the issue. Differences in SFRP4 expression among diagnosis groups was assessed using a series of general linear models along with a Bonferroni adjustment for pairwise comparisons. Possible associations between SFRP4 expression and clinical parameters were assessed using Pearson's correlation coefficient. Disease specific survival and relapse free survival for the expression of SFRP4 were described using Kaplan-Meier curves and statistical significance was determined using Cox regression by computing unadjusted and adjusted hazard ratios. Adjusted Cox models included age categories (≤60, >60), tumor grade (1–2, 3), cancer FIGO stage (I–II, III–IV) and residual disease (<10 mm, ≥10 mm). P-values less than 0.05 were considered statistically significant. Data analyses were generated using SAS software (v9.2, Cary, NC, USA).

## Results

### Loss of SFRP4 expression correlates with aggressive phenotype

**Figure 1:**
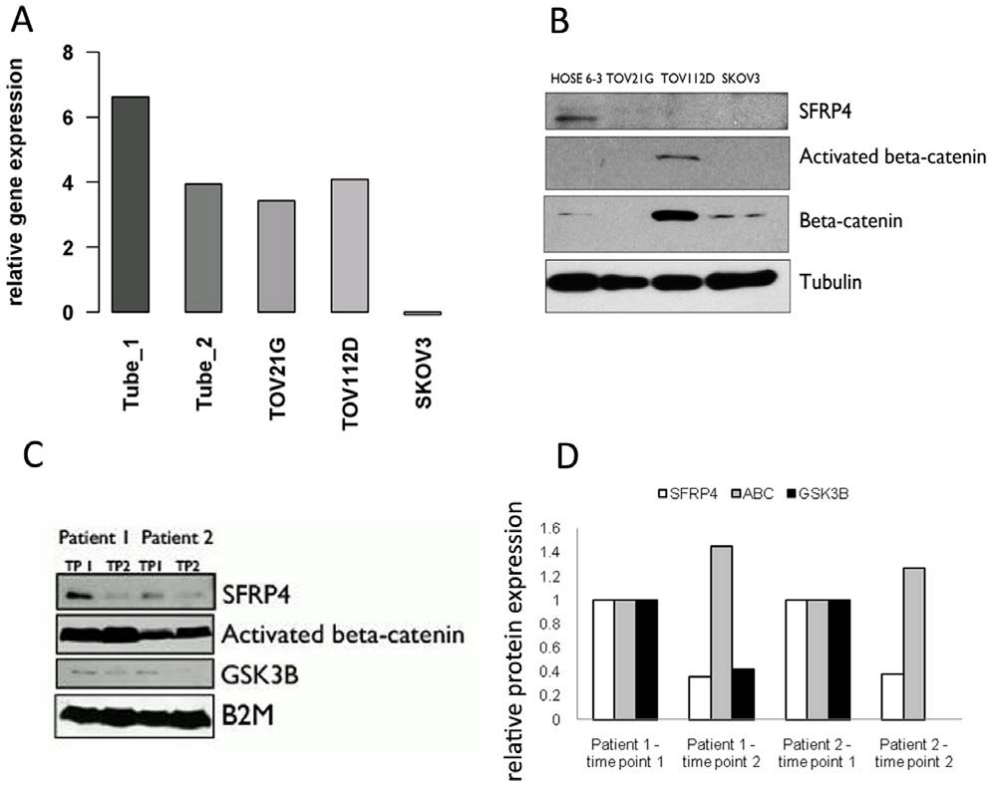
SFRP4 expression in cell lines, primary cultures and ascites. Loss of *SFRP4* expression from primary tubal epithelial cell lines towards ovarian cancer cell lines is shown in various experimental settings. (A) We have applied fluorescence based RT-qPCR to investigate *SFRP4* gene expression in primary tubal (healthy patients) and immortalized cell lines. HOSE6-3 was selected as control and expressions of other cell lines were calculated as a ratio compared to HOSE6-3: R = 2^−[ΔCq*SFRP4*- ΔCqHOSE6-3]^. (B) SFRP4 and its downstream targets activated β-catenin (ABC), β-catenin and GSK3 β were measured by Western-blot in various cell lines (HOSE6-3, TOV21G (clear cell, Type I ovarian cancer), TOV112D (endometrioid) and SKOV3 (serous) Type II ovarian cancers) as well as in (C) ascites samples from high grade serous ovarian cancer patients with chemoresistance, collected at two consecutive time points during disease progression (TP, time point shown as Western-blot; B2M, beta-2-microglobulin was used as loading control). (D) Results for SFRP4, activated β-catenin (ABC), β-catenin, GSK3 β are presented quantitatively using densitometric analysis from one experiment on patient ascites samples.

*SFRP4* was highly expressed in primary tubal cultures when compared to the normal ovarian surface epithelial cell line HOSE6-3 and was particularly high in the patient with the BRCA mutation (Tube 1) as compared to the patient with positive family history of breast/ovarian cancer (Tube 2; Figure 1 A). Whilst a clear cell and endometrioid ovarian cancer cell line (TOV21D, TOV 112D, respectively) expressed *SFRP4* at similar levels to one of the tubal controls, the undifferentiated serous ovarian cancer cell line (SKOV3) displayed very low levels of *SFRP4* (Figure 1 A). SFRP4 expression was then measured at the protein level in various healthy and ovarian cancer cell lines by Western-blot. SFRP4 expression was compared to its key down-stream regulators activated and total β-catenin. HOSE6-3 was compared to various ovarian cancer cell lines of different histotypes and cellular differentiation. SFRP4 expression was lost in all cancer cell lines compared to HOSE6-3 (Figure 1 B). Interestingly, TOV112D, the endometrioid ovarian cancer cell line, expressed highest levels of both activated and total β-catenin, consistent the known Wnt signaling disruption in endometrioid cancers (Figure 1 B) [19]. Ascites fluid collected during progressive chemotherapy resistance was then analyzed for its expression of SFRP4 and downstream targets as a measure of progression over time. Using ascites at two consecutive time points from two cancer patients with supposedly different aggressiveness (Pt 1 mixed ovarian cancer G1, Pt 2 serous peritoneal cancer G3), these experiments showed a reduction of SFRP4 protein expression during progressive chemoresistant disease (Figure 1 C, D). In parallel with SFRP4 decrease, levels of the downstream Wnt signaling regulator GSK3β were also reduced, whereas activated β-catenin increased, suggesting that Wnt signaling was indeed activated in these patients.

### Correlation of SFRP4 tissue expression with various clinicopathological parameters

**Figure 2:**
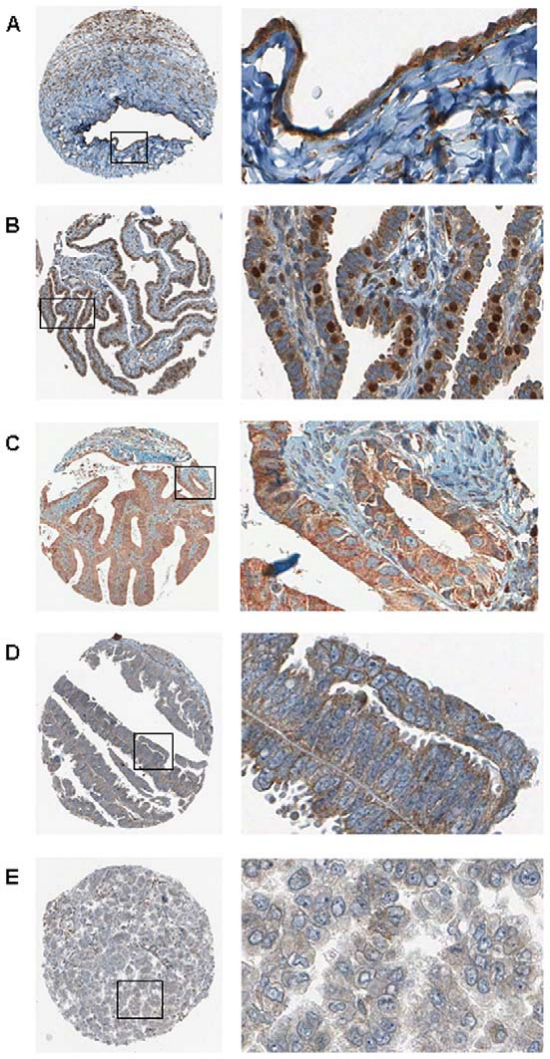
SFRP4 protein expression measured by immunohistochemistry. IHC demonstrating representative SFRP4 protein expression at two magnifications (×10 left column, ×40 right column) in normal tissues (ovarian surface epithelium (A) and tubal epithelium (B)) and various types of ovarian cancers (endometrioid (C), serous (D) and clear cell (E)).

SFRP4 localization was then measured at the protein level due to its proposed function in the membrane, cytoplasm and occasionally also in the nucleus of cells (Figure 2) using IHC in a large cohort of 721 patients on tissue microarrays linked to extensive follow-up data (Table 1). Membranous staining could be identified at the cell-cell boundary and on the apical membrane, which is consistent with its suggested secretory function. Our cohort incorporated 281 control patients who were healthy or had benign conditions like endometriosis or cystadenomas/-fibromas. The cancer group also consisted of 440 patients with borderline tumors or invasive cancers of mostly ovarian (69.8%) but also endometrial (11.3%) and other origins (18.9%). The mean age at diagnosis was 57.1 years (19–88 years) for the whole cohort, with the mean for the control group being five years younger than the cancer cohort (53.7 *vs.* 58.9 years). Our comprehensive database incorporated follow-up data from a maximum period of 29 years (mean for both cohorts 48.4 months (1–348 months)).

SFRP4 was positively expressed in tissue in 296 of 721 patient tissues (41.1%) and in 128 of 142 plasma samples (90.1%). Within the cancer cohort, 279 patients (86.4%) had high grade tumors (grade 3) and 221 patients (55.3%) presented with advanced (FIGO III/IV) stage disease. The five year mortality rate in the whole cancer/Type I/Type II cohort was 29.5/20.6/41.3%, respectively. The five year relapse rate in the same cohort subgroups was 20.5/15.3/27.5%, respectively, thus reflecting a representative cancer cohort.

Possible associations between SFRP4 expression determined both in tissue and plasma, with clinicopathological parameters derived from our in-house clinicopathological database (PEROV database; Access (Microsoft, Seattle USA)) was assessed using Pearson's correlation coefficient, r. Clinicopathological parameters which were accessible within our cohort included abortions, age at diagnosis, BMI, CA125 levels, CA72-4 levels, HE4 levels, grade and stage of the cancers, residual disease, ascites at diagnosis, size and bilaterality of ovarian tumors, performance status at diagnosis, length of follow-up, disease-specific and relapse-free survival, platinum chemotherapy, oral contraceptive or hormone replacement therapies, pregnancies/deliveries, age at menarche and menopause, presence of acne, polycystic ovaries, excess hair, infertility, infections, diabetes, hypertension, alcohol/drug intake, smoking, intake of non-steroidal drugs, past history of hospitalizations, breast disease, endometriosis, fibroids, ovarian cysts, and past history of cancer or familial cancers.

The only moderately strong correlation found for SFRP4 expression both in tissue and plasma was detected in the case of ascites being present at first diagnosis (SFRP4 IHC: r = 0.55, p = 0.01; SFRP4 ELISA: r = −0.60, p = 0.03). Another moderately strong correlation was present for breastfeeding when correlated to SFRP4 plasma expression (r = −0.79, p = 0.06). However, this is of borderline significance as the sample size for breastfeeding was rather small. Weak correlations were found for BMI (SFRP4 IHC: r = 0.19, p = 0.003) and the new tumor marker HE4 (SFRP4 ELISA: r = −0.25, p = 0.02) [34]. A marginally significant relationship could be detected for past history of gynaecological operations (IHC: p = 0.038, ELISA: p = 0.099), hormone replacement therapy (ELISA: p = 0.097) and alcohol intake (IHC: p = 0.098).

### SFRP4 expression is increasingly lost during malignant transformation

As would have been envisaged from its supposed function, SFRP4 expression presented particularly as membrane and cytoplasmic staining. From all tissues examined, only the fimbrial end of the fallopian tube expressed nuclear SFRP4 staining, which was a rather unexpected finding (Figure 2 B). Ovarian surface epithelium, which was until recently proposed to be the uniform place of origin for most ovarian cancers, displayed only minimal cytoplasmic and no membrane staining, as was the case for inclusion cysts, the location of metaplastic changes within the ovary. The highest SFRP4 expression within all studied tissues was found in the tubal epithelium, which is consistent with our findings at the RNA level in primary tubal cultures (Figure 1 A). The newly proposed Type II cancers are increasingly thought to have their origin at the fimbrial end of the fallopian tube, which should then rather be the normal control for most cancers (38). Indeed, we found a decline in cumulative SFRP4 expression from tubal epithelium, benign tissues (including ovarian surface epithelium and inclusion cysts), endometriosis to borderline tumors and cancers (Figure 3.1 A, B), again in consistency with the trend we found by RT-qPCR in cell lines (Figure 1 A). Loss of SFRP4 expression from benign to cancer was statistically significant both when measured as membrane expression alone (p<0.0001 overall, Figure 3.2; Benign *vs.* Cancer, p = 0.07; Borderline *vs.* Cancer, p<0.0001) and when membrane, cytoplasm and nuclear expression were measured in combination (p = 0.0004 overall, Figure 3.1 A; Benign *vs.* Cancer, p = 0.039; Borderline *vs.* Cancer, p = 0.002). Whilst a subdivision of membrane staining was difficult to measure due to small sample sizes in membrane-expressing tissues, the highest expression in the benign group for the total SFRP4 expression was in the tubal epithelium, followed by endometriosis and lowest in ovarian surface epithelium and inclusion cysts (Figure 3.1 B; Tube vs. OSE, p<0.0001; Tube vs. Endometriosis, p = 0.014).

**Figure 3:**
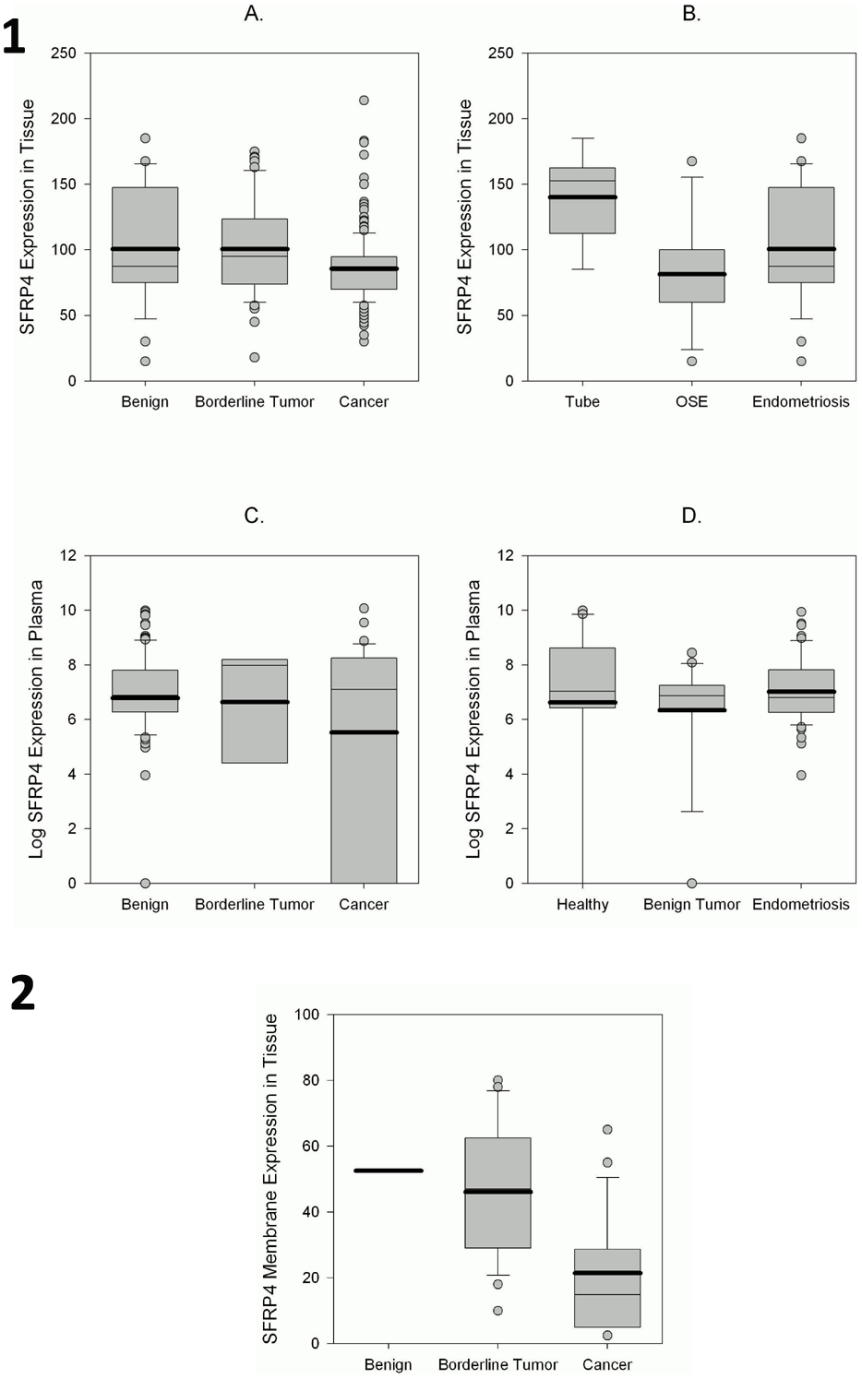
SFRP4 tissue and plasma expression from benign towards cancer. Boxplots representing cumulative SFRP4 expression (arbitrary units) in cytoplasm, membrane and nucleus (SFRP4 Expression in Tissue; 3.1 A) and SFRP4 Expression (Log(SFRP4 Expression) in Plasma; 3.1 C) in plasma of patients with benign diagnosis, borderline tumors and cancers. Subdivision of the benign diagnosis group shows highest tissue expression in tubal epithelium (Tube) compared to ovarian surface epithelium (OSE) and endometriosis (3.1 B) and similar expression levels in the plasma of healthy patients (Healthy) and patients with benign tumors and endometriosis (3.1 D). Membrane-only expression of SFRP4 shows highest expression in benign tissue with a decrease in borderline tumors and a marked reduction in cancers (3.2).

We did not only detect SFRP4 in tissues but also established an ELISA for SFRP4 detection in plasma. Its detection in human plasma, for the first time described here, is consistent with its proposed secretory function, and showed the same trend as in tissues (p = 0.03, Figure 3.1 C; Benign vs. Cancer, p = 0.025).

**Figure 4:**
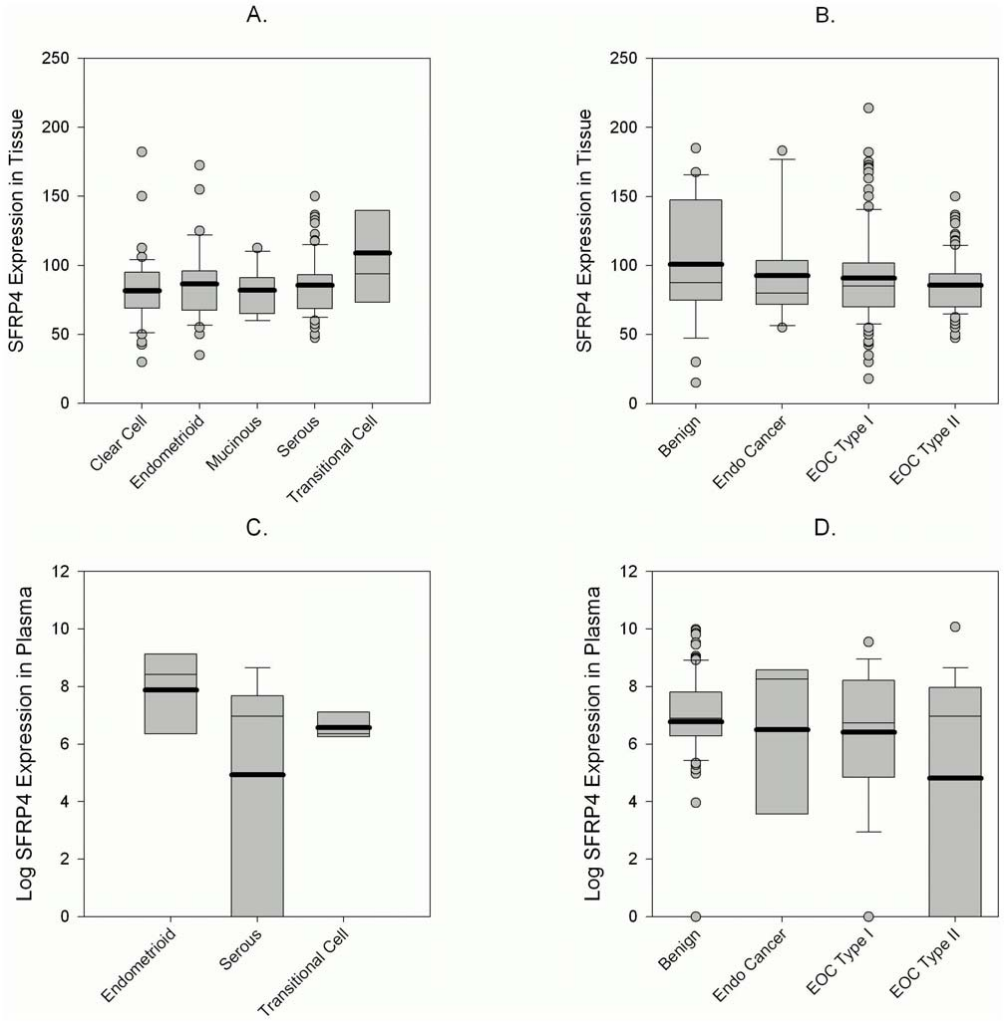
SFRP4 tissue and plasma expression in various diagnoses. Boxplots representing total SFRP4 expression in tissues of various ovarian cancer histotypes shows no significant difference (A). A trend of decreasing expression from benign tumors (Benign) to endometrial cancers (Endo Cancer) and ovarian cancers (EOC) Type II can be seen both in tissue (B) and plasma (D). SFRP4 plasma expression was highest in endometrioid ovarian cancers compared to serous and transitional cell cancers (C).

In contrast to previous literature on endometrioid endometrial cancers, SFRP4 expression was similarly distributed in all histological subtypes of ovarian cancer (Figure 4 A), although in plasma, patients with endometrioid ovarian cancers showed a trend towards higher SFRP4 expression (N.S. p = 0.23; Figure 4 C). Due to reported Wnt signaling defects in endometrioid endometrial cancers we included these adenocarcinomas into our analysis and could demonstrate a lower expression in endometrial cancer patients compared to the control cohort, both in tissue and plasma samples (Figure 4 B & D). When ovarian cancers were split into the newly proposed ovarian cancer Type I and II groups (38), Type II ovarian cancers expressed lower levels of SFRP4, which is consistent with the proposed progression model where Type II cancers have frequent *p53* mutations and present as the more aggressive and rapidly progressing type of ovarian cancer. This trend, however, whilst observed in both tissue (overall and membrane expression) and plasma, was only significant in the later (p = 0.014 overall, Figure 4 D; Benign vs. EOC Type II, p = 0.007)).

No loss in expression could be found between early (I/II) and advanced (III/IV) FIGO stages (p = 0.86; data not shown), but SFRP4 tissue expression decreased with loss of cancer differentiation showing significantly lower levels in undifferentiated compared to well differentiated cancers (p = 0.006 overall; G1 *vs.* G2, p = 0.004; G1 *vs.* G3, p = 0.017; data not shown).

### Loss of SFRP4 membrane expression is associated with poor survival

**Figure 5:**
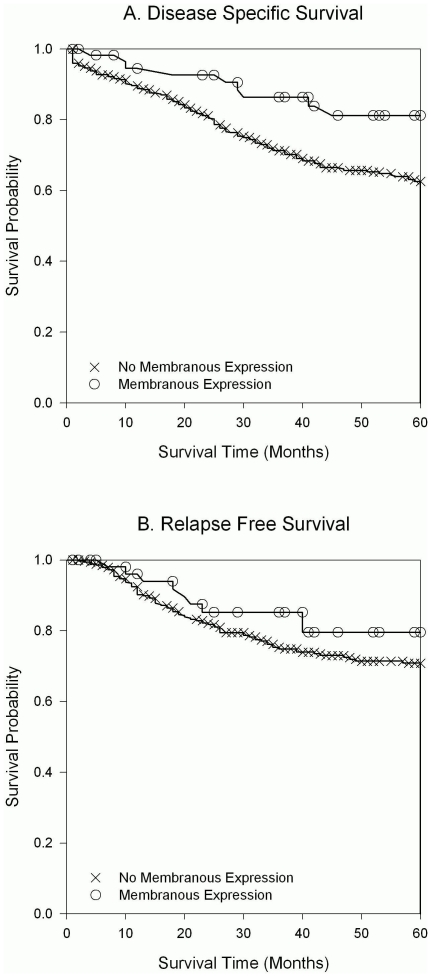
Loss of SFRP4 membrane expression results in poor patient outcome. Kaplan-Meier Curves for disease specific survival (A) and relapse free survival (B) of patients whose tumors have SFRP4 membrane expression (○) or have lost expression (×). Loss of expression is significantly correlated with earlier death of disease as measured using Cox regression by computing unadjusted and adjusted hazard ratios.

Patients with cancers who had lost membrane SFRP4 expression were associated with an earlier death from their disease (p = 0.016, Figure 5 A). This effect was still present (p = 0.089) when SFRP4 was modeled against strong predictors of outcome in ovarian cancer like age, stage, grade and residual disease (p-values 0.0062; 0.014; 0.22; <0.0001, respectively). No similar predictive power was possible for all SFRP4 (combining membrane, cytoplasmic and nuclear staining; p = 0.50) or SFRP4 expression in patient's plasma. The same result could be found when only ovarian/tubal and peritoneal cancers were examined as the number of SFRP4 positive cancers changed only by 3 when other cancers were excluded. Therefore, the effect seen here is clearly ovarian cancer related.

The independent prediction of membranous SFRP4 expression in the 5 year mortality of gynaecological cancer patients has a factor of 1.8, which means that the chance of dying earlier of cancer is 80% higher if SFRP4 membrane staining is absent in their tumors at the time of diagnosis. The power of this prediction within the multivariable model becomes obvious when a strong predictor of prognosis like grade becomes insignificant after adding SFRP4 (p = 0.21). Moreover, the other highly predictive clinical factors like age, stage and residual disease have similar hazard ratios and predictive powers (risk ×0.6 (age), ×2.2 (stage), ×3.2 (residual disease)). No significant prediction was found for relapse-free survival although it showed a similar trend (Figure 5 B).

## Discussion

We previously performed transcription profiling in ovarian cancer specimens compared to normal ovarian controls [3] which revealed aberrant expression of *SFRP1* and *SFRP4* in ovarian cancers. A relationship between down-regulation of *SFRP1* and *SFRP4* and microsatellite instability is known for endometrial cancers. *SFRP4* also suppresses, depending on the receptor, *Wnt-7a*-induced proliferation *via* canonical and non-canonical pathways in endometrial cancer cell lines [35], [36]. Conversely, up-regulation of *SFRP4* was observed for breast, colorectal and prostate cancers which were associated with an increase in cytoplasmic β-catenin levels. Its membrane localization has also been correlated with a good prognosis in various cancers [37].

In this study, we could detect the same survival effect for the first time in a large cohort of adenocarcinomas of gynaecological origin. This effect was present for membranous SFRP4 expression but only had an effect on survival, not on recurrence of the patient. Our experiments contradict a previous small study where SFRP4 expression was measured by IHC in 153 serous ovarian cancers and no survival effect could be found [38]. The advantage of our cohort, however, is the large amount of specimens and the long-term follow-up of 29 years which makes this prediction possible.

We observed the highest SFRP4 expression at both the RNA and protein level in tubal epithelium whilst only low or no membrane expression at all could be found in ovarian surface epithelium. The high protein SFRP4 expression in tubal epithelium was due to membrane, cytoplasmic and particular nuclear staining, which was striking as SFRP4 is only supposed to have an extracellular role. Whilst the low detection rate of membrane staining in ovarian surface epithelium could be due to the thin layer of mesothelial cells, certainly nuclear staining could have been clearly visible but was instead absent. It is unclear at this point why this obvious tubal nuclear staining was observed. Tubal epithelium certainly has an epithelial differentiation, and it is known that overexpression of SFRP4 shifts prostate cancer cell lines toward an epithelial morphology [39], [40]. On the other hand the lowest SFRP4 levels were found in Type II ovarian cancers (advanced stage/undifferentiated serous cancers and carcinosarcoma), which are increasingly thought to be a distinct molecular entity from the less aggressive Type I ovarian cancers [41]. This conjoint finding of highest SFRP4 expression in tubal epithelium and lowest in Type II ovarian cancers fits optimally to the proposed model that Type II cancers derive rather from the tubal epithelium and not the ovarian surface epithelium. The loss of expression not only from borderline tumors towards aggressive phenotype ovarian cancers but also from what is supposed to be the site of origin (fimbrial end of the fallopian tube) towards the cancer also strengthens the proposed putative inhibitory function of SFRP4 within the Wnt signaling pathway. In a recent study, loss of *SFRP5* has been associated with both ovarian carcinogenesis and chemotherapy resistance [29]. Moreover, overexpression of SFRP4 also inhibited proliferation and metastatic potential in prostate cancers and was an independent predictor of outcome [37], [42], [43]. The loss of SFRP4 expression in our study of ovarian oncogenesis supports the results found in other cancers and suggests a tumor suppressor function for SFRP4 also in ovarian cancers. Loss of this function results in a more aggressive phenotype which could be found in patients with progressive chemoresistant disease, again in consistency to reports in other cancers (27).

Apart from confirming an inhibitory effect of SFRP4 in all histotypes of ovarian cancer, this study also demonstrated for the first time that the secreted levels of SFRP4 can indeed be detected in human blood and can therefore be used diagnostically. Whilst SFRP4 plasma expression was not predictive of outcome, its loss of expression from healthy to cancer still supports the findings achieved by RT-qPCR, Western-blot and IHC, and is probably mainly due to lower sample size and shorter follow-up data than our large IHC cohort.

This study suggests that both on gene and protein expression level patients with disrupted function of SFRP4 might be able to be targeted by antagonizing the Wnt signaling pathway. As gene expression of *SFRP* members is often lost through promoter hypermethylation, re-expression of these gatekeepers through the use of epigenetic modifying agents could be one way of antagonizing activated canonical Wnt signaling. The importance of these Wnt gatekeepers has been demonstrated in colorectal cancer, the cancer most commonly associated with aberrant Wnt signaling where re-expression of Wnt antagonists appeared to override strong downstream mutations in APC (adenomatous polyposis coli), and induce apoptosis [44]. Another approach of inhibiting cancers with hyperactivated canonical Wnt signaling, such as ovarian cancer, may be by activating the antagonistic non-canonical Wnt pathway by targeting key receptors or ligands. One gene which could be targeted in this antagonistic pathway is Wnt-5a, which has been shown to act as a suppressor of tumor metastasis in breast cancers [45]. We and others have recently shown that Foxy-5 peptide, a six amino acid fragment based on Wnt-5a, with subsequent modifications has anti-metastatic properties [46].

This study adds to the growing body of literature highlighting the importance of these Wnt gatekeepers in ovarian cancer. It also demonstrates the clinical relevance of SFRP4, a proposed putative inhibitor of the Wnt signaling pathway for both prognosis as well as potentially therapeutic target in ovarian cancer. This is of major importance particularly in the supposedly low risk Type I ovarian cancers where it is crucial to identify patients which will develop aggressive disease despite having a low risk cancer where otherwise adjuvant chemotherapy might not even be administered.
